# Probiotic Bacteria as Therapeutics and Biohybrid Drug Carriers: Advances, Design Strategies, and Future Outlook

**DOI:** 10.1021/acsabm.5c00959

**Published:** 2025-09-01

**Authors:** Deepsundar Sahoo, Edgar Rodriguez, Kytai Nguyen, Uday Chintapula

**Affiliations:** † Department of Bioengineering, 12329 University of Texas at Arlington, Arlington, Texas 76010, United States; ‡ Joint Bioengineering Program, University of Texas Southwestern Medical Center, Dallas, Texas 75235, United States

**Keywords:** probiotics, drug delivery, synthetic biology, nanoparticles, cancer, vaccines

## Abstract

Probiotic bacteria have emerged as versatile and biocompatible platforms for drug delivery, offering a safe and efficient means of targeting diseased tissues. Advances in nanotechnology and genetic engineering have significantly expanded the potential of probiotic bacteria in precision medicine, enabling the delivery of therapeutics, proteins, antigens, and nanoparticles (NPs). This review explores diverse strategies for utilizing probiotics as drug carriers, including bacterial ghosts, outer membrane vesicles (OMVs), surface membrane proteins, and spores, focusing on applications in cancer therapy, vaccine development, and gastrointestinal disorders. We primarily focus on the strategy of integrating probiotics into nanoparticle-based delivery systems, examining key design considerations, such as functionalization strategies, targeting efficiency, and biocompatibility. Additionally, we highlight genetic engineering approaches, including plasmid-based expression and genomic integration, that enhance the probiotic functionality for targeted therapy, immunomodulation, and nanoparticle-mediated drug delivery. Further advancements in synthetic biology, biohybrid coatings, and stimulus-responsive mechanisms that could optimize the therapeutic efficacy of these systems will be discussed briefly. This review comprehensively analyzes recent progress and the outlook for harnessing probiotics for next-generation targeted drug delivery applications.

## Graphical abstract

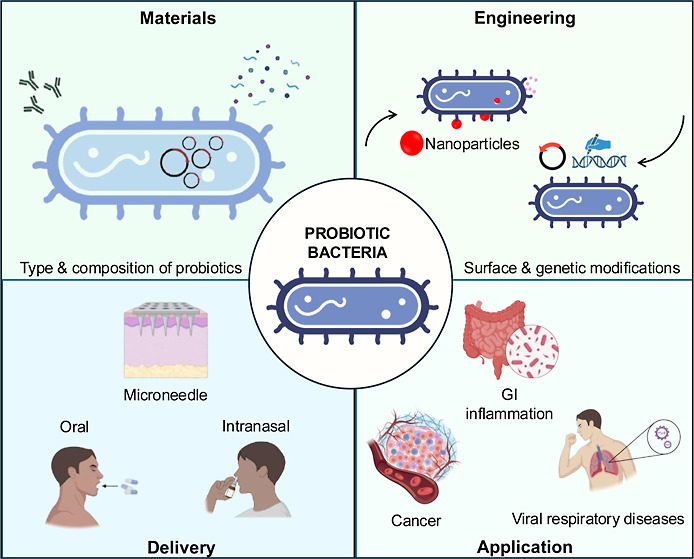

## 1 Introduction

The World Health Organization (WHO) defines probiotic bacteria as microorganisms that confer health benefits when administered in adequate amounts. The concept of probiotics was first introduced by Elie Metchnikoff, a Russian Nobel laureate, in 1906, who reported their potential health benefits. Emerging evidence suggests that live probiotic strains, including major strains of *Bifidobacterium* and *Lactobacillus*, when consumed through food or as supplements, may contribute to gut microbiota homeostasis and promote intestinal health.^1,2^ The U.S. Food and Drug Administration (FDA) classifies most probiotic species, such as the *Lactobacillus* genus, as “generally regarded as safe” (GRAS) due to their long history of safe use in fermented foods and of their presence in the human gut microbiota. According to a global business market research report,^3^ the medical probiotics market is estimated to be valued at $45.76 billion and rise to $70.18 billion in 2034 with a CAGR of 6.3%. The *Lactobacillus Rhamnosus* bacterium market size stood at $1.2 billion in 2024 and is predicted to reach $2.8 billion by 2033, registering a 10.2% CAGR from 2026 to 2033.^4^

Even if the probiotics are viable, certain diseases and their correlations with probiotics are highly debatable. There has been a multitude of research asserting the beneficial effects of probiotic bacteria on cancer, inflammatory diseases related to the human gut, and viral infections.^5^ Probiotics’ primary mechanism of action includes direct hostility against pathogens, reducing bacterial adherence and its invasion capacity in the intestinal epithelium, boosting the immune system, and regulating the central nervous system (CNS).^6^ Additionally, probiotics have specific properties such as resistance to acidic pH, bile tolerance, tolerance to pancreatic fluids, adhesion, and invasion capacity in the intestinal epithelial cells.^7^ Probiotic strains such as *Lactobacillus*, *Lactococcus*, *Bifidobacterium,* and *Bacillus* inhibit the colonization of pathogenic bacteria. Their inhibitory effects result from reducing the luminal pH, competing for nutritional resources, and the secretion of bacteriocins.^8^ Some probiotics also exhibit tumor-specific colonization that can inhibit tumor growth.^9–11^ These cases of using live probiotic bacteria or “living biotherapeutics (LBPs)” are promising for therapeutic applications. However, they are also weighed down by complexities and limitations, such as systemic infections, catalytic decomposition, and the transfer of antibiotic-resistance genes to resident bacteria.^12–14^ To address these limitations, researchers utilize genetic engineering of probiotics and/or their components, such as specific proteins or metabolites, to deliver therapeutic cargos in targeted ways. This strategy, often referred to as “bugs as drugs”, includes innovative applications like engineering bacteria to produce insulin directly in the gut to treat diabetes. One of the significant ways to do this is by integrating the bacterial surface with a myriad of biological moieties, producing multiple functionalities through their biological actuation and sensing capabilities. A few “bug as a drug” therapies are being investigated in clinical trials, but their market availability has yet to be seen.

Recent advancements in nanotechnology-based drug delivery systems (DDS), particularly with nanoparticles (NPs), have enabled precise targeting for medical imaging, diagnostics, and therapeutics. However, challenges, such as inefficient targeting, rapid clearance, and toxicity, persist. Probiotic bacteria present a promising alternative due to their biocompatibility and capability to synthesize and transport nanoparticles. Despite their potential, their role as nanoparticle delivery vehicles remains largely untapped. This review examines the therapeutic value of probiotics in cancer, vaccine, and GI disorder applications, current strategies for targeted delivery of NPs using probiotics, various design considerations, and their limitations.

## 2 Therapeutic Applications of Probiotics and Their Components

Current studies increasingly aim to elucidate the specific interactions of probiotics with gut microbiota and other biological systems to modulate the human immune system and clarify the mechanisms through which they confer therapy. The notion of a “bug as a drug” refers to genetically engineered or recombinant bacteria that, when delivered into the body, can navigate directly to diseased cells or tissues, resulting in specific therapeutic outcomes. Probiotics have been genetically modified to express a range of compounds, such as bacterial toxins, RNAi, small molecules, immunomodulating peptides, and other enzyme products that act as prodrugs.^15,16^ A popular example is the generation of insulin in *Escherichia coli* bacteria for diabetes treatment. In this case, *E. coli* was not directly used as a carrier for insulin but as a “biofactory” intended to produce substantial amounts of insulin. The probiotic “bug as drug” approach has been used in treating cancer, viral infections, and metabolic and autoimmune diseases.^17^ Probiotic bacteria have emerged as versatile LBTs with relevance across seemingly distinct disease contexts owing to their ability to modulate host immunity, localize to specific tissues, and serve as safe, modifiable carriers for therapeutic payloads. Similarly, their components such as spores, surface layer material, outer membrane vesicles (OMVs), bacterial ghosts, and other relevant material, combined as “living therapeutic materials (LTMs)”, are also employed in engineering therapies. In cancer, certain probiotics can influence tumor-associated immunity and can be engineered to deliver anticancer agents directly to tumor sites. In vaccine development, their intrinsic immunostimulatory capacity and mucosal colonization enable them to act as oral or mucosal adjuvants and antigen delivery vectors.^17^ In inflammatory bowel diseases, probiotics contribute to restoring microbiome balance, reducing inflammation, and delivering localized therapeutics with reduced systemic toxicity. These three main areas of oncology, vaccines, and gut inflammatory disorders represent pathologies where probiotics’ safety profile, immune modulation, and site targeting can be leveraged in distinct yet mechanistically connected ways, justifying a focused discussion on these indications over other disease areas such as cognitive health. Various probiotic materials employed for the design of therapeutics are shown in Figure 1. In this perspective, we have critically discussed the role of probiotics and their components as therapeutics and drug carriers.

**Figure 1:**
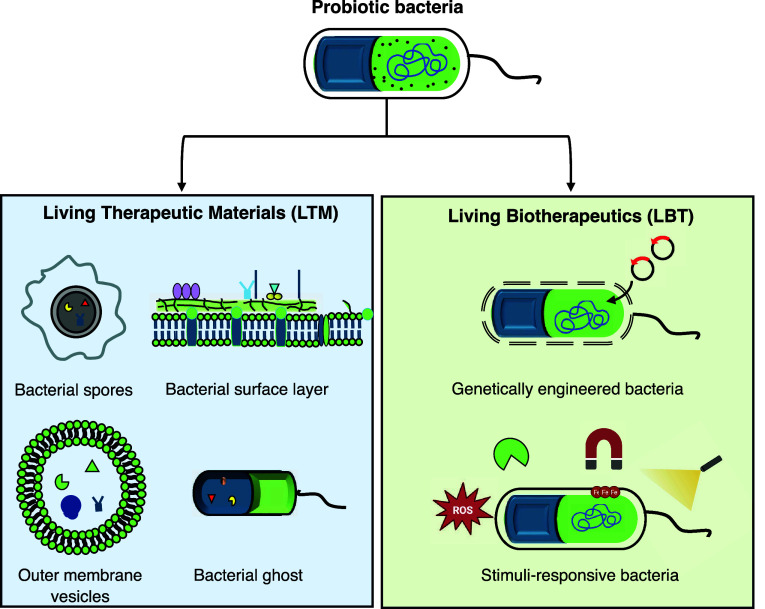
Schematic depiction of probiotic bacteria and their components used for therapeutics. Components are divided into Living Therapeutic Materials (LTMs) and Living Biotherapeutics (LBTs).

### 2.1 Cancer Therapy

#### 2.1.1 Bug as Drug

Among bacteria, probiotic bacteria are a class of nonpathogenic bacteria gaining attention because of their ability to invade the cancer tissue precisely due to hypoxic conditions, modulate cancer cells’ proliferation and apoptosis, as seen both in vitro and in vivo. Colorectal cancer (CRC) is often associated with intestinal microbial dysbiosis.^18^ Probiotic bacterial strains can restore gut microbial homeostasis via gut microbiota modulation. A study revealed that the oral delivery of *Lactobacillus plantarum*, a probiotic originating from fermented milk, suppressed tumor growth by increasing CD8+ and natural killer (NK) cell infiltration and promoted Th1-type CD4+ T differentiation and IFN-γ levels in the CT26 subcutaneous-tumor mouse model.^19^ Use of checkpoint-blocking immunotherapy combined with the probiotic bacteria provided synergistic effects against cancer.^20,21^ Similarly, a study revealed that the combinative administration of *Lactobacillus acidophilus* lysates and cytotoxic T-cell lymphocytes-associated protein 4 (CTLA-4) blocking antibodies inhibited colon carcinogenesis in azoxymethane/dextran sulfate sodium AOM/DSS syngeneic BALB/c mice.^19^ Other experiments on mice have demonstrated the vital role of gut microbiota (*Bacteroides* and *Bifidobacterium*) in anti-PD-L1 (programmed death-ligand 1) and anti-CTLA-4 therapies.^22,23^ An early clinically controlled and comparative study demonstrated that combination therapy, which included radiation therapy alongside heat-killed *Lactobacillus casei* (LC9018) strain probiotics, aided in improving the induction of immune response mechanisms against cancer cells. This immunomodulation enhanced the deterioration of tumors in 223 patients with uterine cervical carcinoma.^24^ Native probiotic strains such as *L. casei* and *L. plantarum*, among others, have been shown to have therapeutic effects, emphasizing the feasibility of probiotic use as a “bug as a drug” in cancer therapeutics.

#### 2.1.2 Engineered Probiotics

While utilization of the native probiotic strains alone as therapeutic agents is promising, the therapeutic capacity of each bacterium is limited to a certain extent. Engineered probiotics have been widely used to deliver cytotoxic proteins, prodrug-converting enzymes, angiogenesis regulation proteins, RNAi molecules, and immunoregulatory factors. One study employed a genetically modified *E. coli Nissle* 1917 strain as a vector designed to express toxic protein HlyE, and this bacterium preferred to be colonized in tumor tissues inducing tumor regression in mice xenografted with human CRC cells.^25^ RNAi is known to be an efficient gene-silencing technology, making it a potential tumor gene therapy tool to silence cancer-inducing genes. However, RNA interference (RNAi)-based therapies face challenges related to insufficient tissue targeting and degradation. Bacteria-mediated RNAi therapy involves employing the probiotic as a vector for the transportation of RNAi effectors such as short hairpin RNA (shRNA) into the target cells and silencing the target mRNA and eventually the gene expression. For instance, in a study, the role of attenuated bacteria such as *S. typhimurium* as a potential vector for delivering synthetic RNA has been investigated.^26^ This strategy showed that the bacteria engineered with a plasmid (pSLS) constructed with the hlyA gene and T7 RNA polymerase gene could express shRNA for silencing specific genes. The modified *S. typhimurium* (SL-pSLS-huCAT) displayed a significant reduction of colorectal carcinoma-inducing gene (CTNNB1) expression both *in vitro* and *in vivo*. This knockdown further inhibited tumor growth and reduced the size and number of polyps in the mice bearing SW480 xenograft tumors and APC^Min^ mice.^26^ These studies show a way to overcome the limitations of the therapeutic capacity of probiotic bacteria in their native state.

Engineering approaches could utilize bacterial strains such as *E. coli Nissle*, *Bifidobacterium* species, and *Lactobacillus* species that can survive gastric acid and bile salts and colonize the gut mucosa.^27^ However, probiotics have the potential to mutate and evolve undesirable traits during the diagnosis, leading to the loss of beneficial functions of the engineered system and gain of detrimental functions, such as the competitive exclusion of native microorganisms, pathogenic potential to the host, or environmental contamination. Therefore, biological containment strategies such as (i) use of plasmids without antibiotic resistance genes,^28,29^ (ii) auxotrophic strains,^30,31^ (iii) orthogonal system-based biological containment,^32,33^ (iv) passive suicide circuits,^34^ (v) physical containment,^35^ and (vi) induced suicide circuit^36,37^ should be considered to provide a kill switch before any harmful mutations cause imbalance and related side effects in the host GI tract flora.

#### 2.1.3 Outer Membrane Vesicles and Bacterial Ghosts

Similar to engineered probiotic bacteria, their components, such as outer membrane vesicles (OMVs) and bacterial ghosts (BGs), due to their targeting abilities, have also been investigated for drug delivery. In one study, chemically modified ghosts were derived from Gram-positive probiotic lactic acid bacteria functionalized with prodigiosin (PG), a cytotoxic secondary metabolite against cancer cells.^38^ In vitro results of the study from treatment of colorectal cancer cell lines (HCT116) revealed that the PG ghosts significantly upregulated the p53 protein level, which frequently mutates or is silenced in CRC. Bacterial OMVs are composed of nonreplicating bilayer membrane that can deliver chemotherapeutic drugs, nucleic acids, and immunotherapeutic agents. For instance, hypervesiculating *E. coli Nissle* releasing OMVs packed with Cytolysin A (ClyA)-Hyaluronidase (Hy) at the tumor site resulted in a reduction of hyaluronic acid synthesis and smooth muscle actin of tumor tissues.^39^ Additionally, in vivo data demonstrated that delivering the hypervesiculating probiotic combined with PDL1 antibody significantly suppressed tumor growth and improved survival in MC38 tumor mice by enhancing the therapeutic antibodies and facilitating immune cell infiltration.^39^ However, the systemic toxicity caused by PAMPs in OMVs presents a significant challenge for their clinical translation.^40–42^ A recent study showed that calcium phosphate (CaP)-based surface mineralization of the OMVs synthesizing melanin (OMV (mel)) intracellularly helped reduce their toxicity and enhance antitumor efficacy^43^ (Figure 2). The laser-activated calcium phosphate shell disintegration from the OMVs activates the OMVs for their photothermal-induced melanin release, triggering an antitumor response. In comparison with the nonmineralized OMVs, they showed lower inflammatory responses and less damage in the organs of mice. Use of the OMVs and bacterial ghosts can enable custom engineering for personalized medicine and reduce the complexity of biological interactions from a live bacterium.

**Figure 2:**
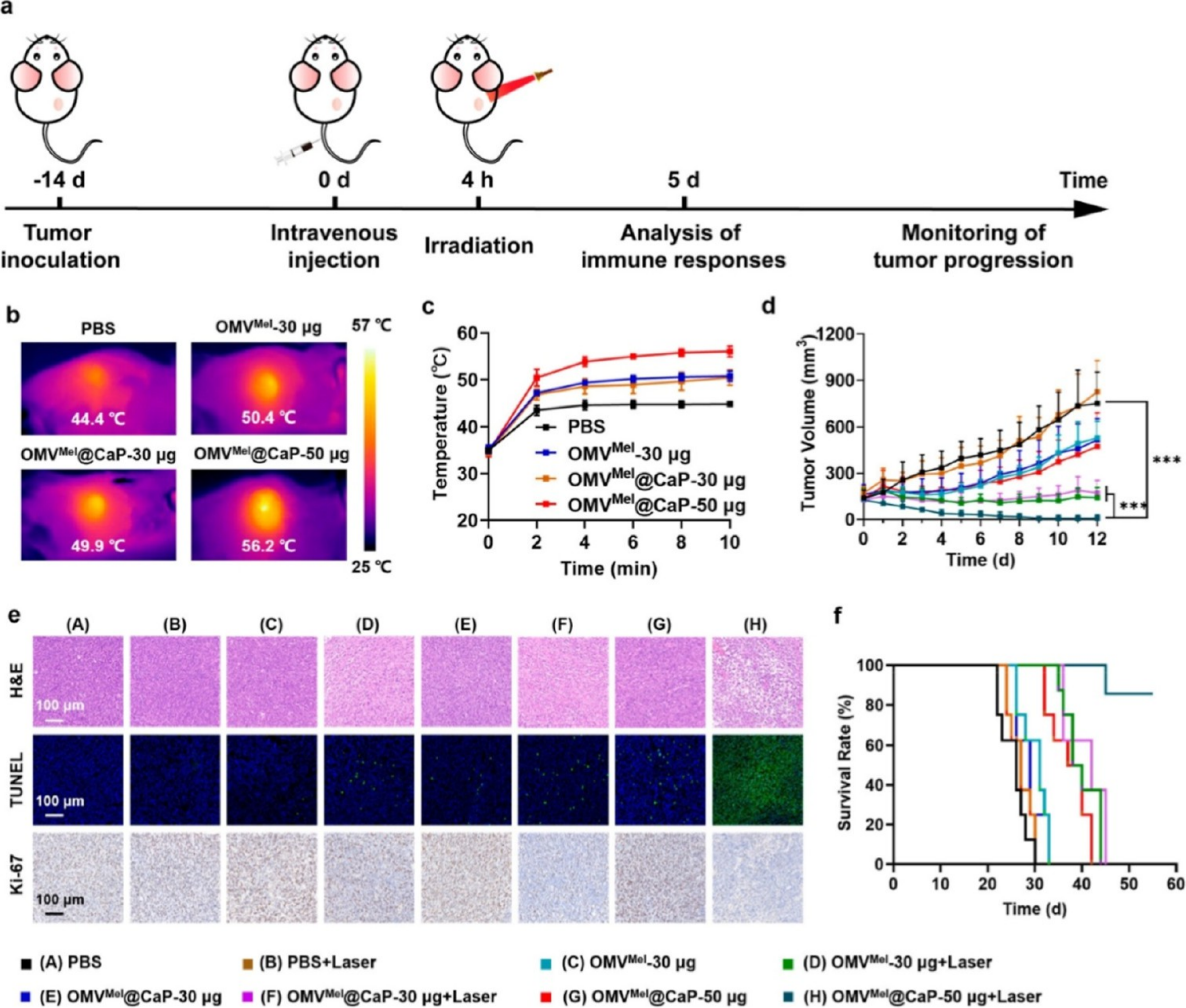
In vivo antitumor efficacy of melanin-OMV@CaP. (a) Schematic diagram for antitumor photothermal immunotherapy in 4T1 tumor-bearing mice via intravenous injection. (b) Infrared thermal image and (c) heating curve of mice treated in different ways (*n* = 3). (d) Tumor volume growth curves monitored for 12 days (*n* = 6). (e) Staining of H&E and TUNEL, and *K*_i_-67 fluorescence of main organs. TUNEL^+^ cells were green, and *K*_i_-67^+^ cells were brown. (f) Survival period of tumor-bearing mice in each group (*n* = 8).^43^ Copyright © 2024, American Chemical Society. Reprinted (Adapted) with Permission from Xue Chen, Puze Li, Ban Luo, Cheng Song, Meichan Wu, Yuzhu Yao, Dongdong Wang, Xuyu Li, Bo Hu, Suting He, Yuan Zhao, Chongyi Wang, Xiangliang Yang, and Jun Hu. *ACS Nano* **2024** *18*(2), 1357–1370.

#### 2.1.4 Spores

Spores, or resistant cells seen in bacteria and other major animal kingdoms, have been engineered to present an anticancer prodrug and are being utilized for the delivery of chemotherapeutic drugs to cancer cells.^44,45^ To overcome the adverse side effects of the intravenous (IV) administration of most chemotherapeutic drugs, a study utilized autonomous generating nanospores derived from the probiotic *Bacillus cagulans* containing Doxorubicin (DOX) and Sorafenib (SOR) through oral administration.^46^ These spores were surface-decorated with Deoxycholic acid (DA), which could increase the internalization of the spores postendocytosis. Oral administration of the spores in the rats bypassed the harsh gastric environment due to their surface coating by a thick hydrophobic protein layer, which resists harsh acidic conditions. Also, it demonstrated that the drug-containing spores could germinate and autonomously generate nanospores in the acidic tumor microenvironment without requiring additional external driving force, subsequently taken up by the intestinal epithelium, with enhanced apoptosis and necrosis, indicating significant antitumor activity.^46^

Overall, the use of LTMs, containing lipopolysaccharides, poses minimal concern from an oral therapeutic standpoint. However, it may have significant safety implications when administered systemically or intratumorally in the translational phase. In some cases, minimal toxicology studies may be needed if the agent is not disseminated from a local site.^47^ Alternatively, the use of LTMs could provide several advantages as a drug carrier in comparison to the use of the LBTs. OMVs display excellent biocompatibility and enhanced capabilities for membrane modification owing to their nanoscale dimensions and distinctive spherical lipid bilayer vesicle structure for stability. BGs are a bacterial shell with a porous structure, which is convenient for loading antitumor drugs and does not contain any genetic material, effectively mitigating the risk of horizontal gene transfer. Spores also offer exceptional stability and ease of storage. Due to these distinctive biological attributes, LTMs can serve as highly efficient drug delivery vehicles, significantly enhancing antitumor efficacy.^48^

### 2.2 Viral Infections

#### 2.2.1 Immune Modulation Mechanisms

Viral infections have become more common in recent decades, posing a significant threat to human health.^49–54^ The clinical application of the mRNA–lipid NP vaccine against viral respiratory infection significantly improved people’s lives by protecting them against the virus.^55^ A probiotic-bacterial-based delivery system represents a next-generation biomimetic platform, offering the advantage of safety while employing synthetic biology approaches to combat these lethal infections. Targeted engineering can make these probiotics appropriate as a vector to transfect nucleic acids, proteins, and genetic materials into the host cells.^56–58^ The primary mechanism of the probiotic bacteria’s action against infections has been explained mainly by three biological processes, such as antimicrobial activity, support of the epithelial barriers, and immunomodulation (Figure 3).^59^ When the microorganism-associated molecular patterns (MAMPs) present on the cell surface of bacteria engage dendritic cells (DC) pattern-recognition receptors (PRRs), the DCs mature and migrate toward the lymphoid tissues, leading to CD80 and CD86 upregulation and MHC II expression, effectively performing antigen presentation to T-cells. This T-cell activation also drives a balanced pro-inflammatory cytokine milieu such as TNF-α, which aids in the DC activation and the antigen presentation within a threshold to prevent its systemic overproduction to cause tissue injury. The IL-6 cytokine, driving both pro-inflammatory and anti-inflammatory pathways, is upregulated during the infection but is down-regulated by probiotics to prevent cytokine overshoot. DC-driven regulatory CD4+ T-cells also secrete TGF-β and IL-10, which are strongly upregulated due to the probiotic–DC interactions acting as an anti-inflammatory mediator, promoting the integrity of the epithelial barrier, inducing tolerance, and resisting inflammation. This coordinated response aids in the restoration of Th1/Th2 homeostasis and may enhance the CD8+ cytotoxic T-cell in the lungs for rapid viral clearance while also reducing the severity of cytokine storm. Probiotic interventions can act as adjuvant modulators, improving mucosal and systemic immunity.

**Figure 3:**
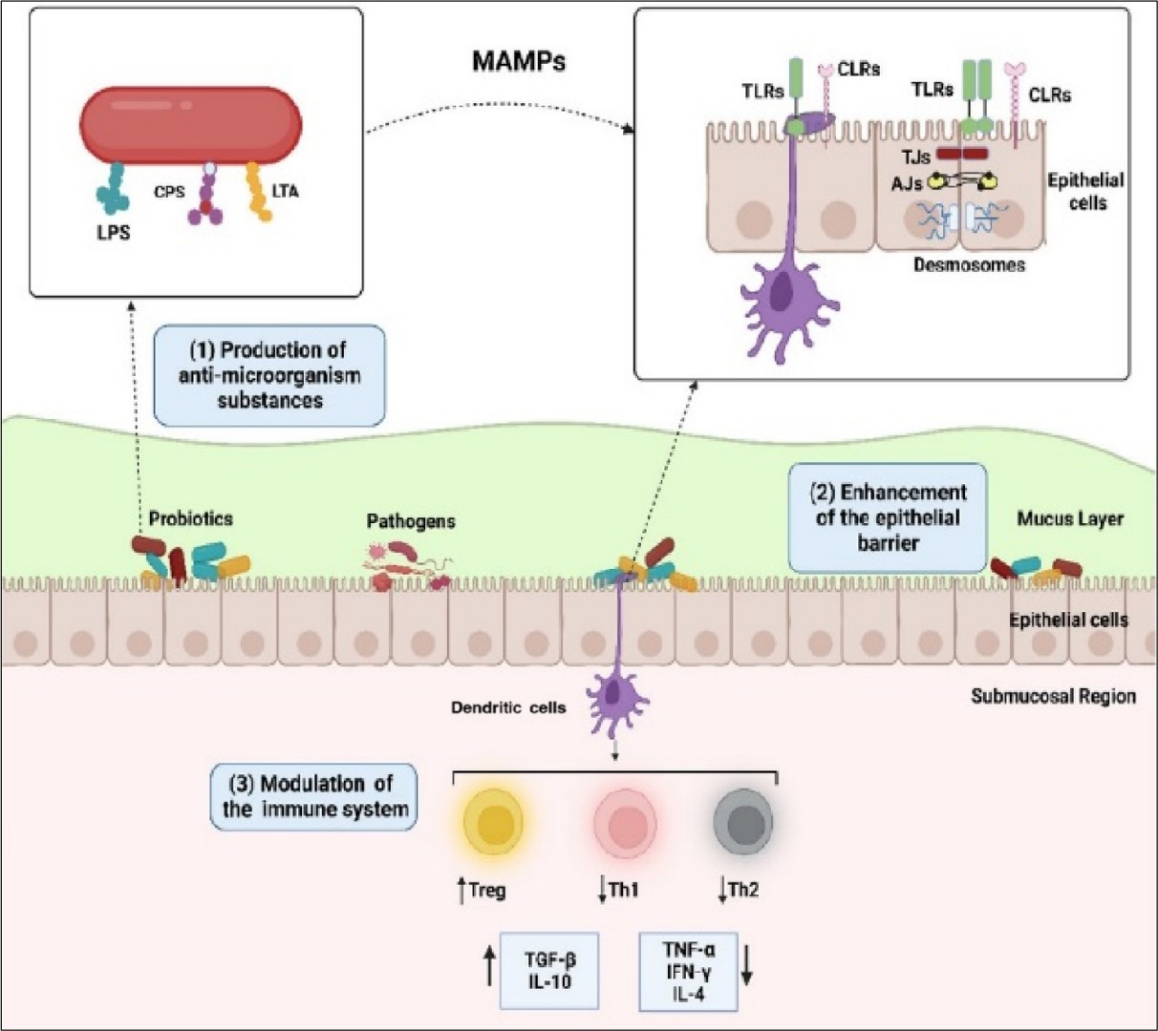
Primary mechanisms of action of probiotics against infections. (1) Probiotics produce antimicrobial substances like bacteriocins that cause cell death by inhibition of pathogen cell wall synthesis, (2) can enhance the barrier properties of epithelium enhancement of epithelial barrier by an interaction between MAMPs (i.e., LPS, CPS, and LTA) on the surfaces of probiotics and pattern recognition proteins on the epithelial barrier or modulation of intercellular junctions such as TJs, AJs, and desmosomes, and (3) can modulate the immune responses by interacting with dendritic cells. LPS: lipopolysaccharide; CPS: cell-wall-associated polysaccharide; LTA: lipoteichoic acid; MAMPs: microorganism-associated molecular patterns; TLRs: toll-like receptors; CLRs: C-type lectin receptors; TJs: tight junctions; AJs: adherence junctions.^59^ Copyright ©2023 The authors. Published by the American Chemical Society. Reprinted (Adapted) with permission from Nilufer Yuksel, Busra Gelmez, and Ayca Yildiz-Pekoz. *Molecular Pharmaceutics* **2023**, *20*(7), 3320–3337 DOI: 10.1021/acs.molpharmaceut.3c00323.

Recent studies suggest that probiotic bacterial strains, such as *Bacillus*, activate TLR3 in alveolar macrophages, mimicking viral infections and inducing a synergistic immune response with increased TNF-α and IL-6 expression.^60^ Probiotic L.*rhamnosus* administered nasally in mice reduced tissue damage by modulating immune responses, while certain probiotic strains interacted with the SARS-CoV-2’s ACE-2 receptor, releasing ACE-inhibitory peptides.^61,62^ In another study, engineered probiotic *Lactobacillus paracasei F19* expressing *N*-acyl phosphatidylethanolamine-specific phospholipase D (pNAPE-LP) homed to the lungs post-intranasal delivery, preserving the alveolar structure and sharply reducing neutrophil infiltration, myeloperoxidase activity, and histological injury in the lungs of C57BL/6J mice challenged intranasally with SARS-CoV-2 spike protein.^63^ This was achieved by mitigating TLR4-mediated NLRP3 activation and the downstream pro-inflammatory products such as ILs, TNFα, C-reactive protein, and the myeloperoxidase activity. Interestingly, a global reduction in ACE2 expression in the lungs was observed, as well.

#### 2.2.2 Engineered Probiotics for Antiviral Therapeutics

With the increasing popularity of targeted drug delivery systems, bacteria have been utilized in various ways, and the distribution methods employed by bacteria-driven vaccines and therapeutics primarily include membrane vesicles, bacterial ghosts, surface displays, and lysates. It has been revealed that OMVs from the *E. coli Nissle* and other *E. coli* strains deliver mediators that trigger host immune and defense responses.^64^ These vesicles are internalized by intestinal epithelial cells via clathrin-mediated endocytosis and sorted to lysosomes through endocytic compartments.^64^ In addition, OMVs can be decorated with foreign peptides or proteins utilizing bio-orthogonal click chemistry techniques. In one study, it was reported that bacterial OMVs displaying an influenza A-based peptide (ClyA-M2e4xHet) gave maximum protection from a lethal challenge with H1N1 and H3N2 virus strains, subject to administration in a mouse model.^65^ In another recent study, OMVs (NR-OMVs) derived from probiotic bacteria *E. coli Nissle* 1917 were engineered with two different strains of SARS-CoV-2 antigens, receptor-binding protein (RBD) of the spike protein in the OMV’s lumen and NG-06, the fragment of RBD on the surface, as a bivalent antigen display platform.^66^ In vivo data revealed that the NR-OMVs could induce humoral immune responses, increase IgG titers, and enhance immunogenicity.

Chemically induced BGs are also considered as vaccine candidates against viral infections.^67,68^ The presence of intact surface structures in BGs enhances their immunogenicity, therefore eliciting robust immune responses. Yu et al. developed BGs using the probiotic *L. casei* to explore its potential as a novel DNA delivery system.^69^ The study showed that BGs with plasmid VP6 from Porcine Rotavirus significantly upregulated IL-1β, IL-10, TNF-α, arginase-1, iNOS, CD 206, TLR-2, TLR-4, and TLR-9 in macrophages. M1 (IL-10, TNF-α) and M2 (Arg-1, CD 206) polarization markers were also increased. In vivo, IgG levels were higher in serum, with T-cell polarization toward Th1, essential for combating viral infections. BG-based therapeutics also enhanced the CD4+/CD8+ T-cell ratio, indicating a balanced immune response. LTMs driven by antiviral responses and drug delivery could be harnessed to utilize these microorganisms as ingestible adjuvants for immune modulation and strengthening the vaccine-induced memory responses against acute/chronic viral infections.

### 2.3 Inflammatory Gastrointestinal Disorders

Chronic inflammation of the gastrointestinal (GI) tract is a common characteristic of inflammatory bowel disease (IBD), a chronic idiopathic disease including Ulcerative Colitis (UC) and Crohn’s disease (CD).^70^ These intestinal disorders involve chronic inflammation of the gut lining, followed by a series of events including barrier disruption, immune overactivation, and gut microbial imbalance. The vulnerable epithelial barrier allows the luminal bacteria and antigen penetration, aiding in overinflammation and perpetuating tissue damage.^15,71^ Probiotic bacteria offer health benefits against chronic GI tract disorders by actively counteracting IBD through coordinated and complex pathways to ensure the restoration of microbial balance and reinforce the gut barrier (Figure 4).^72^ LBT strains are well suited for colonization of the GI tract with the ability to produce therapeutic benefits in situ. The use of probiotics provides an alternative that rebalances the gut microflora, shifting the balance from a proinflammatory to an anti-inflammatory state. They mechanistically modulate the gut microbiome composition by exclusion of the infectious pathogens through the creation of an acidic environment, production of antimicrobial peptides,^73,74^ and metabolites that inhibit the growth of pathogenic microbes and compete with the intestinal microbes^75^ for binding sites on the intestinal mucosal surface (see Figure 4).^73–75^ Moreover, probiotics confer additional benefits through microbial enzymatic activities, such as Bile salt hydrolase (BSH) enzymes that deconjugate biliary salts, thereby promoting bile acid metabolism.^76^ Probiotics are also reported to modify intestinal immunity through altering the responsiveness of intestinal epithelia and immune cells. Probiotic strains such as *Lactobacillus sakei* and *Lactobacillus johnsonii* can reduce intestinal inflammation by downregulating TLRs through interference with enterocyte signaling pathways (such as NF-κB and TNF-α secretion), as well as by modulating the expression of pro-inflammatory cytokines, thereby skewing immune responses toward regulatory, wound-healing phenotypes.^77,78^

**Figure 4:**
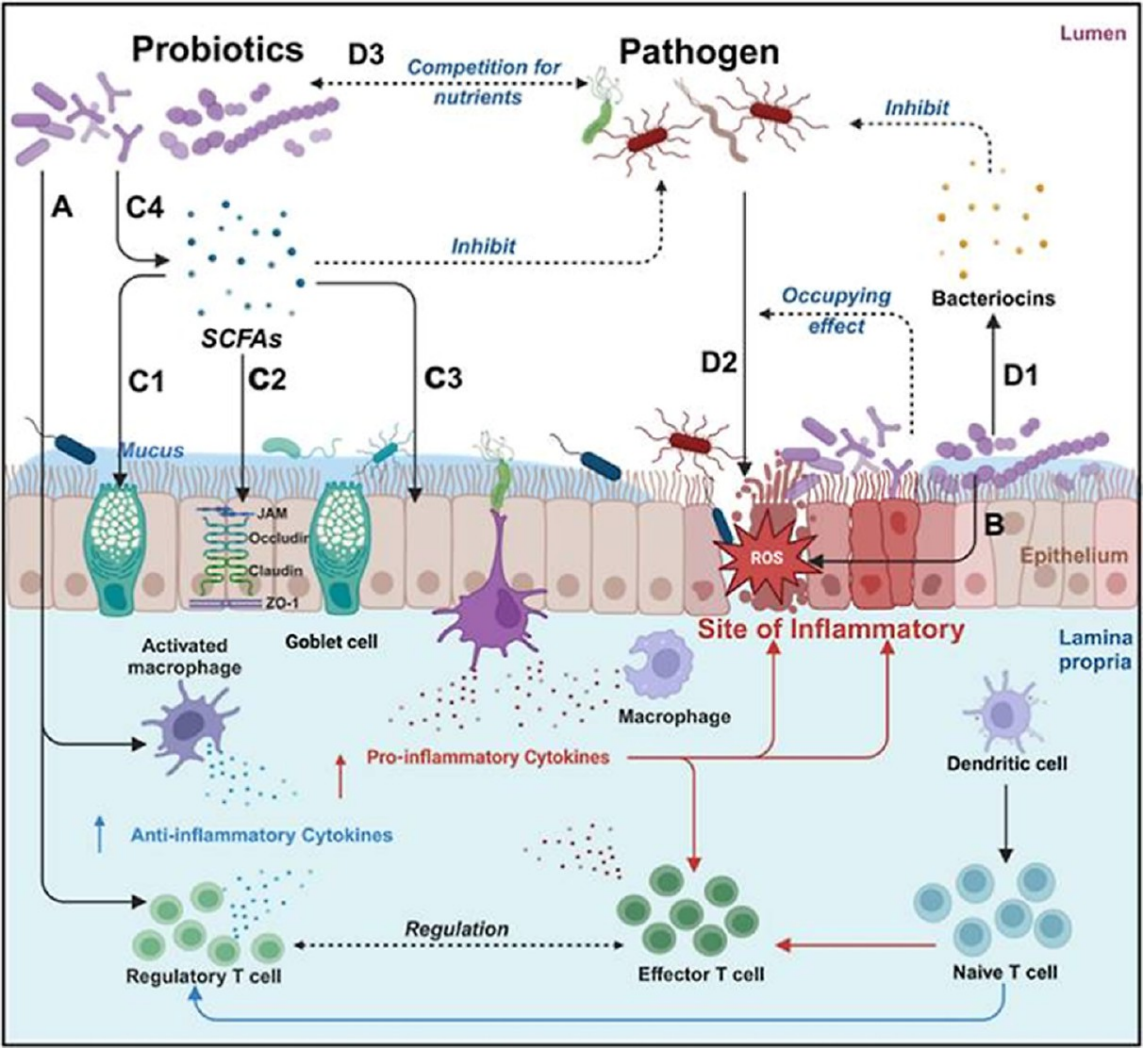
Overview of the main mechanism of probiotics in the treatment of IBD. Probiotics have various therapeutic effects on IBD; different probiotics may have different degrees of therapeutic effects; they can be roughly divided into immune regulation, antioxidant, anti-inflammatory, repairing intestinal barriers, and helping the body resist pathogenic microorganisms. A: Immunomodulation: promotion of anti-inflammatory factor expression and inhibition of pro-inflammatory factor expression; B: Neutralization of ROS; C: Probiotics can upregulate mucus protein secretion by goblet cells (C1), enhance tight junction protein function (C2), and intestinal epithelial cell function (C3) by secreting SCFAs (C4); D: Probiotics can inhibit pathogenic bacterium growth by the secretion of bacteriocins (D1) and reduce pathogenic bacteria adhesion through occupancy effect (D2).^79^ Copyright © 2025 The Authors. Published by Theranostics. Reprinted (Adapted) with Permission from Sang, G., Wang, B., Xie, Y., Chen, Y., & Yang, F. *Theranostics* **2025**, *15*(8), 3289–3315. DOI: 10.7150/thno.103983.

Engineered probiotic bacteria enable the development of robust strains with enhanced functional properties, allowing for the targeted control of pathogenic microbes and specific interventions for inflammatory bowel disease (IBD). Probiotic bacteria are designed as factories to produce one or multiple therapeutic biomolecules, with genetically engineered probiotics expressing therapeutic proteins in an inducible manner preferred over a constitutive manner, as it allows for easier control of biomolecule production and prevents overdosing.^80^ In an experimental study, the administration of IL-10-secreting *Lactococcus lactis* induced a 50% reduction of colitis in DSS-treated mice and prevented the onset of colitis in IL-10­(−/−) mice. Mechanistically, IL-10 controls IFNγ-secreting CD4+ T cells in humans and identifies IL-1β as a potential classifier for a subgroup of IBD patients. This approach may lead to a better method for cost-effective and long-term management of IBD in humans.^81^ Similarly, it was found that engineered IL-27-producing *L. lactis* proved more effective than both the IL-10-producing counterpart and systemic administration of IL-27 in colitis mouse models.^82,83^ Treatment with IL-27 attenuates experimental colitis through the suppression of IL-17-producing helper T-cells in the TNBS-induced colitis model, even after active colitis was established. These results suggest potential treatment approaches for IBD, including Crohn’s disease and ulcerative colitis.^84,85^ Other than LBTs, LTMs of probiotic bacteria such as OMVs,^64,86,87^ spore coats,^88^ and s-layer proteins^89^ have also been investigated for the treatment of inflammatory GI disorders.

## 3 Probiotic Bacteria as a Nanoparticle Delivery System

The discovery of nanotechnology has significantly advanced the drug delivery field; however, several limitations and challenges must be addressed to achieve efficient drug delivery.^90,91^ Nanoparticles still rely on the leaky vasculature for passive drug delivery, which has very low delivery efficacy, leading to off-target side effects in cancer treatment.^92^ Most of the nanoparticles, such as lipids, polymers, and inorganics, are still mainly cleared by organs such as the liver and spleen. Researchers have focused on precision therapeutic strategies to overcome these challenges, including multimodality-based drug delivery systems, cell/gene therapy, bacterial therapies, and other vaccine-based strategies. Among these approaches, probiotic-bacteria-mediated therapy could be considered a promising strategy with the potential to significantly impact the field of therapeutics. Most of the probiotic bacteria are safe and nonpathogenic, with high scalability. Probiotic bacteria can be engineered for carrying NPs, either via encapsulation, surface-conjugated, or as factories synthesizing NPs.

### 3.1 Live Probiotics Carrying Nanoparticles

To address off-targeting and cellular uptake problems, NPs (organic and inorganic) have been incorporated within the bacterium or conjugated on its outer surface, which serves as an efficient vehicle for the delivery of NPs. These cargo-carrying bacteria, also termed “microbots or Bacteriabots”, aid in colonizing impenetrable regions such as tumors and deliver therapeutics. An early research study reported that various particle-size (40 nm vs 200 nm in diameter) polystyrene (PS) NPs loaded with nucleic acid molecules were noncovalently attached to the surface of attenuated *L. monocytogenes* for delivery in in vitro and in vivo models.^93^ When compared to in vitro cellular uptake with NPs alone, it was found that bacterial-mediated NPs (200 nm) were successfully delivered after 3 h of incubation. In vivo data demonstrated that microbots delivered the gene into mouse organs, expressing proteins at levels 380-fold higher than those of the PBS controls. Similarly, probiotics were used for the delivery of Poly­(propylene sulfide) (PPS), a hydrophobic polymer known to scavenge ROS. Due to its hydrophobicity, the clinical application of the polymer is limited.^94,95^ In a recent study, self-assembled hyaluronic acid–PPS-conjugated NPs, amphiphilic in nature, were conjugated to the surface membrane of the probiotic *E. coli Nissle* that was coated with Norepinephrine (NE)^96^ (Figure 5). NE was utilized to enhance the oral delivery of probiotics by the formation of biofilm on the bacterial surface and protecting it from the harsh external assaults of the GI tract. When the *N*P-conjugated probiotics were orally administered to the induced mouse model, they exhibited improved resistance toward extreme conditions, enhanced retention time, improved survival rate, and enhanced prophylactic and therapeutic effects to alleviate the inflammation.^96^

**Figure 5:**
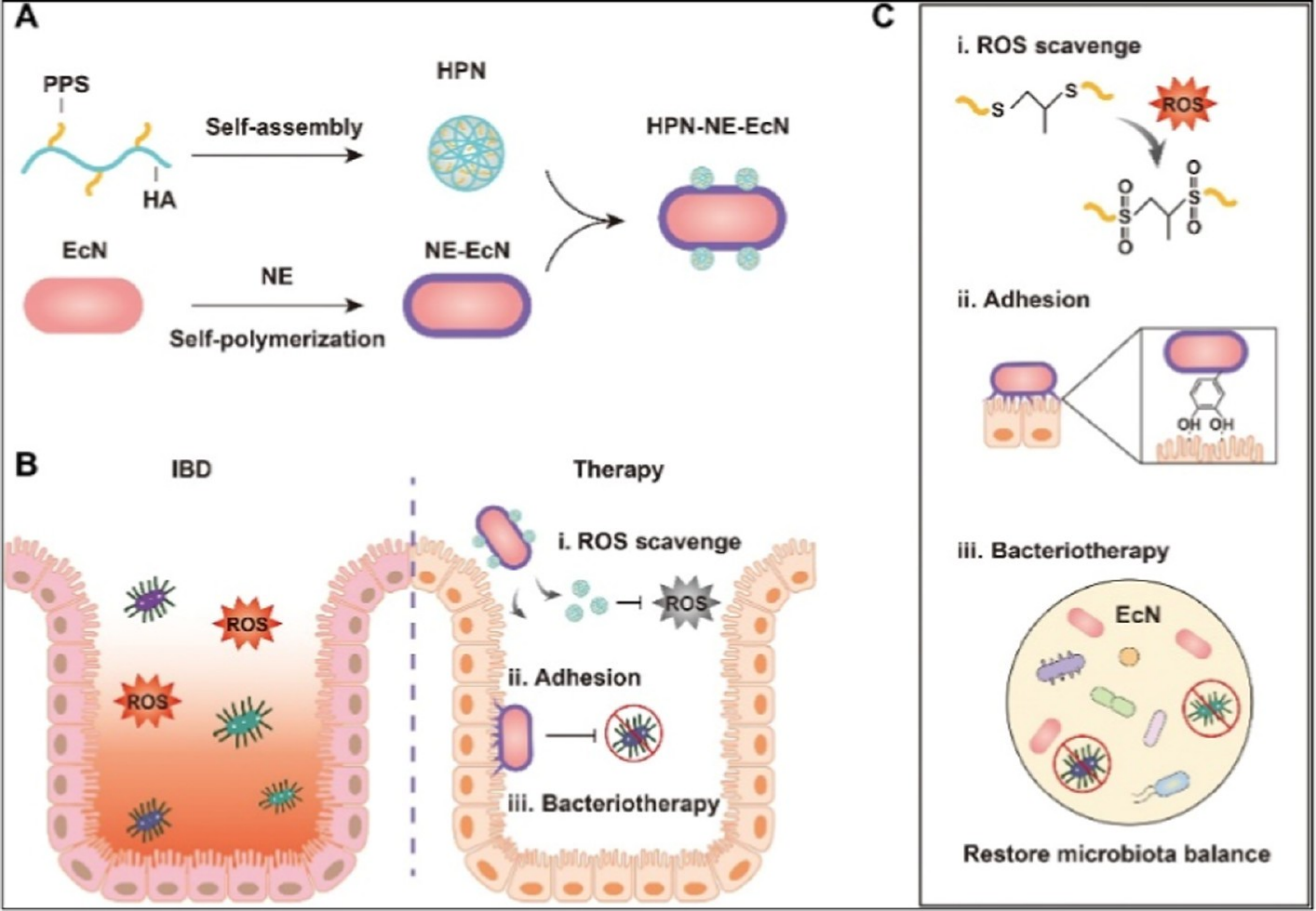
Schematic illustration of the preparation of HPN-NE-EcN and its mechanism for IBD treatment. (A) Preparation of HPN by self-assembly of the HA-PPS molecule, encapsulation of *Escherichia coli* Nissle 1917 (EcN) with the norepinephrine (NE) layer, and conjugation of HPN to the surface of EcN. (B and C) The prepared HPN-NE-EcN exerts ROS-scavenging activity by oxidizing sulfur atoms in PPS to form sulfoxides and then further oxidizing to form sulfones (i). Furthermore, the NE layer, which mimics mussel adhesive foot proteins, endows EcN with a strong mucoadhesive ability and extends the retention time of EcN in the intestine (ii), allowing for enhanced bacteriotherapy through restoring the gut microbiome homeostasis (iii).^96^ Copyright ©2022, The American Association for the Advancement of Science. Reprinted (adapted) with permission from Jun Liu, Yixin Wang, William John Heelan, Yu Chen, et al. *Science Advances* **2022**. DOI: 10.1126/sciadv.abp8798.

Engineered magnetic particle-carrying bugs that are externally driven by propelling magnetic forces can aid in targeted delivery in harsh physiological environments. Toward that approach, in recent studies, probiotic bacteria have been engineered to be used as platforms to either load or decorate surfaces with superparamagnetic NPs (MNPs). In one study, a biohybrid microbot able to be driven by magnetic fields, thermal conditions, and hypoxic environments utilized surface-conjugated magnetic nanoparticles with *E. coli Nissle* to provide a stable magnetothermal switch under an alternating magnetic field.^97^ The authors demonstrated that the electrostatic attachment and encapsulation of MNPs on the bacterial surface does not significantly affect the bacterial surface compared to controls without any MNP encapsulation. Furthermore, the probiotic’s viability under the magnetothermal conditions was acceptable, as shown by the flat colony counting method and bacterial growth curve in their research. This was achieved by an optimized magnetic field applied for inducing a localized and reversible heat shock response without affecting the bacterial cell viability. The temperature-sensitive bacteriophage λ repressor class c1857 (Tcl) in bacteria activates NDH-2 enzyme expression in response to temperature changes, increasing the level of H2O2 and mCherry for fluorescence imaging. In vivo imaging demonstrated that microbots effectively targeted and penetrated tumors using spatial magnetic coordination and hypoxia sensing. Similarly, a biohybrid micro-robotic system of genetically engineered *E. coli* expressing biotin-attaching peptides and GFP enhanced outer functionalization^98^ (Figure 6). The genetically engineered bacteria were surface-functionalized with ICG-DOX carrying stimulus-responsive nanoliposomes for on-demand delivery of the therapeutics and magnetic NPs for external swimming control and navigation of microbots in the tumor matrix. This biohybrid model demonstrated enhanced penetration into the tumor cells and localized and external stimulus-triggered on-demand release of anticancer therapeutics in a 3D tumor spheroid model. These applications show the engineering ability of stimulus-responsive probiotics via nanoparticle interactions.

**Figure 6:**
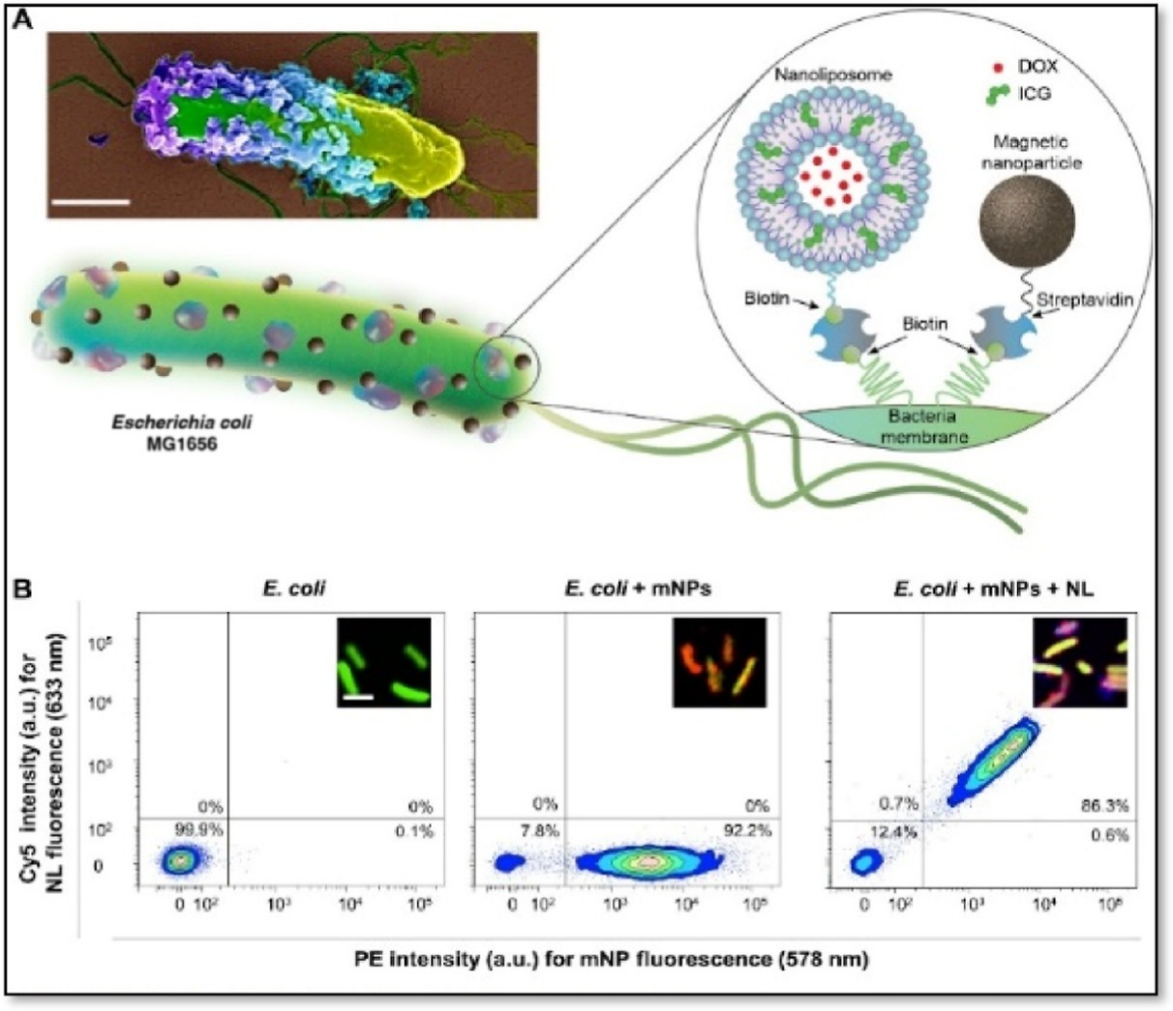
Bacterial biohybrids carrying magnetic NPs (mNPs) and Nanoliposomes (NLs). (A) Schematic illustration of the bacterial biohybrid microrobots, conjugated with NLs and mNPs. NLs are loaded with DOX and ICG, and both NLs and mNPs are conjugated to bacteria via biotin–streptavidin interactions. The inset shows an SEM image of an example bacterial biohybrid carrying mNPs and NLs. The image is pseudo-colored. Scale bar, 500 nm. (B) Flow cytometry density plots of (i) free bacteria expressing GFP, (ii) bacterial biohybrids carrying mNPs tagged with red fluorescence, and (iii) bacterial biohybrids carrying mNPs tagged with red fluorescence and NLs tagged with Cy5, showing successful conjugations quantitatively. a.u., arbitrary units. Scale bar, 2 μm.^98^ Copyright © Reprinted with permission Mukrime Birgul Akolpoglu et al. Magnetically steerable bacterial microrobots moving in 3D biological matrices for stimuli-responsive cargo delivery. *Sci. Adv*. **2022**, *8*, eabo6163.

Quantum dots (QDs) have created a particular interest as a nanoparticle that could be employed as a multifunctional tool for diverse in vitro and in vivo applications. Despite its numerous advantages, its pitfall of being intrinsically toxic and inflammatory toward normal cells has hindered its approval from being a drug payload and imaging agent.^99,100^ Liu et al. developed a microbot-based payload system using an anaerobic probiotic bacterium *Bifidobacterium Bifidum* that could deliver the QDs specifically colonizing the hypoxic site of the deep tumor region for tumor detection through in vivo imaging.^101^ Folic acid (FA) conjugation on the bacteria aided the microbot toward the tumor region by binding to the folic acid receptors overexpressed at the tumor sites. The QDs were encapsulated in the membrane of bacteria through electroporation. It was hypothesized that the probiotic bacteria, being anaerobic, have the potential to settle deep in the tumor region and release the QDs. This demonstrates the strategic use of probiotics to deliver toxic compounds in a biocompatible manner.

The bacterial surface can also be engineered as a drug-loading site, enabling multimodal, stimulus-responsive delivery systems. This strategy offers advantages such as spatiotemporal control over drug release and the ability to incorporate magnetic or optical guidance, which can enhance penetration through dense extracellular matrices in tumor-mimicking environments. However, loading drugs externally on the bacterial surface introduces certain challenges. Drug molecules are exposed to enzymatic degradation, hydrolysis, or premature release in circulation, potentially reducing therapeutic potency before reaching the target. Moreover, surface-bound drugs may interact nonspecifically with host cells or serum proteins, leading to off-target effects or immune activation. These risks necessitate careful consideration of drug–bacteria conjugation chemistry, protective coatings, and controlled-release linkers.

### 3.2 Intracellular Biosynthesis of NPs

The physical and chemical synthesis of metallic nanoparticles for drug delivery faces significant challenges, including low production yield, contamination-induced toxicity, functionalization issues, and stability concerns.^102,103^ To address these limitations, alternative synthesis methods have been explored to minimize the toxicity and side effects of chemical compounds in biological systems. One such approach involves the intracellular biosynthesis of inorganic nanoparticles using bacteria, which has been extensively studied for its applications in diagnostics, imaging, and therapy. Bacterial systems can serve as biofactories for synthesizing various nanoparticles, including gold, silver, platinum, palladium, titanium, titanium dioxide, magnetite, and cadmium sulfide. In one study, probiotic strains of *Lactobacillus* spp. (*L. plantarum* and *Lactobacillus fermentum*) were used as carriers for biosynthesized cadmium sulfide (CdS) nanoparticles.^104^ These nanoparticles demonstrated efficient uptake at tumor sites and exhibited a high tolerance within MCF-7 human breast carcinoma cells. Another study reported that *L. casei* ATCC 393 facilitated the synthesis of biogenic selenium nanoparticles, which were found to protect against intestinal epithelial barrier dysfunction and oxidative stress by maintaining epithelial permeability in cells exposed to H_2_O_2_.^105^ In vitro experiments revealed that these biogenic nanoparticles exhibited strong antioxidant activity, low cytotoxicity, and improved mitochondrial function in human colon mucosal epithelial cells.

Additionally, probiotic bacteria can modulate the toxicity of heavy metal nanoparticles. For instance, *L. acidophilus* has been shown to convert toxic Se^+IV^ or Se^+VI^ into nontoxic selenium nanoparticles.^106^ In another study by Mirjani et al., *L. plantarum* was utilized to biosynthesize tellurium nanoparticles in a less toxic and biocompatible form, which effectively mitigated hypercholesterolemia, a major risk factor for coronary artery disease and atherosclerosis.^107^

Gold nanoparticles (AuNPs) have also gained attention as therapeutic carriers due to their biocompatibility and efficacy in drug delivery. Their optical properties enable multimodal imaging and stimulus-responsive drug release upon laser irradiation. A study demonstrated that biosynthesized gold nanoparticles loaded with ginsenoside compound K, synthesized via an intracellular membrane-bound mechanism in *Lactobacillus kimchicus* (isolated from Korean kimchi), enhanced both the photothermal and chemotherapeutic effects against various cancer cell lines.^108^ At a concentration of 5 μg/mL, these bacteria-incorporated nanoparticles significantly inhibited cancer cell growth in various organs while minimizing toxicity in normal cell lines. Overall, probiotic bacteria present a viable platform for synthesizing metallic nanoparticles with enhanced therapeutic potential while mitigating the toxicity commonly associated with conventionally synthesized metallic nanoparticles. A list of bacterial strains and their intervention in nanoparticle-mediated drug delivery is listed in Table 1.

**Table 1:** Probiotic Bacteria as a Nanoparticle Delivery System

bacterial strain	types of NPs (P-payload; B-biosynthesis)	associated disease conditions	synthesis techniques	experimental models used	reference
Lactobacillus plantarum	iron-pectin NP (P)	iron deficiency	surface-conjugated via glycosidic linkage	in vitro digestion assays in various simulated solutions	^109^
Bifidobacterium animalis	biosynthesized gold NPs (B)	anti-inflammation therapy	co-cultured, followed by incubation	in vitro (LPS-induced RAW 264.7 macrophages) in vivo (LPS-induced C57BL/6 mice; 3 groups)	^110^
Roseburia Intestinalis	magnetic iron oxide NP (P)	Crohn’s disease (CD)	co-precipitation method/nonspecific adsorption	in vitro (human colon epithelial cell line NCM460). In vivo (colitis-induced male Sprague–Dawley rats; 6 groups)	^111^
E. coli Nissle 1917	mesoporous silica NPs (P)	intestinal tumor	EDC-NHS chemical cross-linking conjugation	in vitro (human colon cancer cells HCT-116). In vivo (HCT-116 tumor-bearing BALB/c mice; 4 groups)	^112^
L. Plantarum	biosynthesized selenium NP (B)	breast cancer	co-cultured, followed by incubation	in vivo (4T1 tumor-induced BALB/c female mice; 2 groups)	^113^
E. coli Nissle 1917	hyaluronic acid-poly(propylene sulfide) NPs (P)	inflammatory Bowel diseases	surface conjugated via ROS-responsive linker with a terminal amine group	In vivo (DSS-induced colitis in female mice; 6 groups)	^96^
Bifidobacterium bifidum	quantum dots micelles (P)	tumor targeting and imaging	encapsulated via the electroporation method	in vivo (tumor-induced male C57BL/6N mice)	^101^
L. Kimchicus	biosynthesized gold NP (B)	detection of apoptosis	co-cultured, followed by incubation	in vitro analysis in various cell lines	^108^
Lactobacillus rhamnosus	chitosan, hyaluronic acid, ononin NPs (P)	bacterial pneumonia	surface conjugation via electrostatic interaction	in vitro analysis in various cell lines	^114^
E. coli Nissle 1917	gold NPs (photosensitizer) (P)	breast cancer	dual pH-sensitive amide and imine bond conjugation	in vitro (MCF-7 cancer cell lines). In vivo (MCF-7-induced female BALB/c mice; 6 groups)	^115^
L. Plantarum	tellurium NP (B)	hypercholesterolemia	co-cultured, followed by incubation	in vivo (PTU-induced BALB/c mice; 5 groups)	^107^
Bifidobacterium infantis	poly(ε-caprolactone)-mPEG NPs (P)	lung cancer	surface-conjugated via a polydopamine linker	in vitro (A549 cancer cell lines). In vivo (A549-tumor bearing mice; 5 groups)	^116^
E. coli Nissle 1917	magnetic particles (P)	gastrointestinal diseases	surface conjugation via a biotin-streptavidin linker	in vivo (Female BALB/c mice)	^117^
Lactobacillus casei	biosynthesized selenium NP (B)	intestinal barrier dysfunction	co-cultured, followed by incubation	in vitro (NCM460 human colon mucosal epithelial cell lines)	^105^
Bifidobacterium	mesoporous silica NPs (P)	dysbiosis/Gut microbiota imbalance in Alzheimer’s	co-precipitation method, followed by surface modification; co-culture and glutaraldehyde fixation	in vivo APP/PS1 mice model	^118^

## 4 Design Considerations for Probiotics-Based Drug Delivery Systems

### 4.1 Nanoparticle Conjugation Approaches

Bacterial entry into mammalian host cells involves a variety of mechanisms. Harnessing the unique properties of probiotic bacteria for NP delivery represents a transformative approach to drug delivery, overcoming limitations associated with conventional nanoparticle-based therapies. Probiotics, being inherently biocompatible and nonpathogenic, offer a safe and effective means of transporting therapeutic payloads while evading rapid immune clearance. Their natural ability to navigate complex physiological environments enables targeted delivery to diseased sites, such as tumors and inflamed tissues, enhancing therapeutic precision. Advances in surface functionalization techniques, including covalent conjugation, electrostatic interactions, and ligand-based binding, allow for stable NP attachment, improving drug retention and controlled release. Moreover, biohybrid systems integrating stimulus-responsive mechanisms, such as magnetic guidance, light-triggered activation, and pH-sensitive drug release, further refine delivery efficiency by ensuring site-specific activity.

To understand the effect of different pH conditions on the successful delivery of probiotics, one study utilized layer-by-layer polyphenol NP-modified E. coli Nissle 1917, and its resistance against GI tract assaults was investigated in vitro.^119^ The authors showed that the layer-by-layer NP modification protects the probiotic from acid damage and improves survival under simulated gastric fluid (SGF) conditions (pH: 1.2). Additionally, after treatment with simulated intestinal fluids (SIF) (pH: 6.8) and bile salts containing trypsin, the system displayed that the NPs-modified probiotic surface provides resistance toward the enzymatic degradation. The evaluation of NP-coated probiotics toward ROS in the hydrogen peroxide environment displayed ROS scavenging potential, enabling IBD microenvironment remodeling as well as promoting the probiotics’ viability and achieving colonization in the injured part of the colon and more effectively in different pH conditions. Although these innovations hold great promise, scalability, and long-term stability, regulatory considerations remain critical factors for clinical translation. Some key considerations for probiotic engineering as a drug carrier are listed in Box 1.
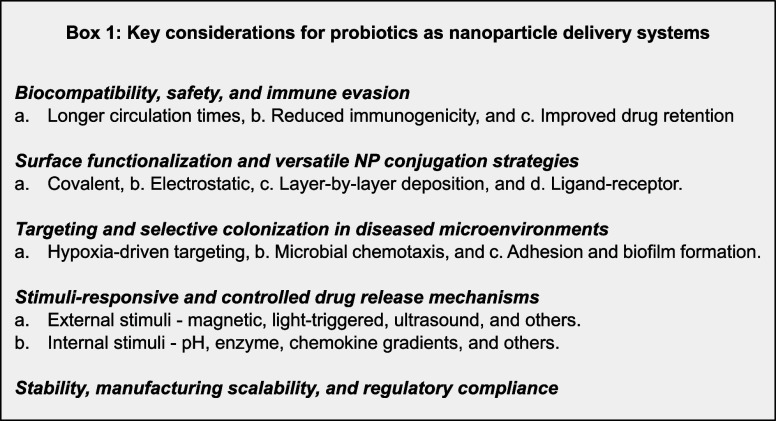


### 4.2 Genetic Engineering Approaches

Synthetic biology revolutionizes microbial engineering by designing and constructing genetically programmed systems to modulate biochemical pathways through gene cloning, protein/peptide overexpression, and metabolomic alterations (Table 2). By integrating nanotechnology, artificial intelligence (AI), and post-translational modifications, synthetic biology enables the development of precise, targeted, and responsive probiotic therapeutics.^120^

**Table 2:** Design of Genetically Engineered Probiotic Bacteria and Their Components for the Treatment of Diseases

probiotic bacteria strains	bacterium/bacterial components	genetic modification	expressed therapeutics	disease	ref.
E. coli Nissle 1917	live engineered bacteria	chromosomal insertion for quorum sensing	cholera autoinducer 1 (CAI-1)	cholera	^139^
L. casei 334, L. acidophilus 4356	S-layer protein	plasmid expression	surface layer protein A (SlpA); a host cell-binding domain from C. difficle SlpA	clostridium difficile infection (CDI)	^140^
E. coli Nissle 1917	OMVs	genome-integrated expression	SARS-CoV-2 RBD (surface); NG-06 (lumen)	SARS-CoV-2	^66^
L. lactis NZ9000	hydrogel-encapsulated bacteria	plasmid expression (NISIN)	VEGF	diabetic wound healing	^141^
E. coli Nissle 1917	live engineered bacteria	plasmid expression	curli nanofibers displaying trefoil factors (TFFs)	inflammatory bowel disease	^80,142^
E. coli Nissle 1917	live engineered bacteria	plasmid expression	l-arabinose-inducible protein expression	solid tumor/cancer	^143^
E. coli Nissle 1917	live engineered bacteria	plasmid expression	p53 and Tum-5 protein	solid tumor/cancer	^144^
E. coli Nissle 1917	live engineered bacteria	genome-integrated expression	GM-CSF cytokine	colorectal neoplasia	^145^
E. coli Nissle1917	chitosan/sodium alginate-coated engineered bacteria	plasmid expression	catalase and superoxide dismutase (ECN-pE)	inflammatory bowel disease	^146^
L. reuteri	live engineered bacteria	plasmid expression	IL-22	ovarian cancer	^147^
L. lactis	live engineered bacteria	genome-integrated expression	IL-10	inflammatory bowel disease	^31^
E. coli Nissle 1917	S-layer protein	CRISPR-Cas9 genome editing	HIV-1MPER	HIV infection	^148^
E. coli Nissle 1917	live engineered bacteria	CRISPR-Cas9 genome editing	type I-E CRISPR-Cas	antimicrobial resistance	^149^
L. Lactis F15876	live engineered bacteria	plasmid expression (NISIN)	GLP-1 peptide	diabetes	^150^
E. coli BL21DE3	live engineered bacteria	plasmid expression	short hairpin RNA (shRNA)	gene silencing	^151^
E. coli Nissle 1917	live engineered bacteria	genome-integrated expression	l-arginine	hyperammonemia	^152^
E. coli Nissle 1917	live engineered bacteria	plasmid expression	Microcin H47 (MccH47)	salmonella infection	^153^
E. coli MG1655	live engineered bacteria	plasmid expression	glucose dehydrogenase	colorectal cancer	^154^

A well-engineered probiotic bacterium must fundamentally consist of (i) regulatory input signals-environmental or host-derived stimuli that activate specific genetic circuits; (ii) genetic circuitry-engineered genetic networks ensuring precise control over gene expression and function; and (iii) output signals with theranostic utility, expression of therapeutic proteins, immune modulators, or metabolic alterations for disease treatment and diagnostics.^120^ Synthetic biology allows probiotics to mimic pathogen surface receptors, neutralize toxins, and enhance immune responses, enabling pathogen-specific targeting and therapeutic intervention. The integration of computational tools and AI further aids in optimizing strain design, predicting biochemical interactions, and improving efficacy and biosafety in clinical applications. Plasmid encoding and genomic integration systems are mainly employed for the genetic engineering of probiotic drug delivery systems.

#### 4.2.1 Plasmid-Encoded Expression Systems

Plasmid-based genetic engineering remains a widely used method for recombinant protein production due to its simplicity and flexibility. The gene expression level is controlled by plasmid copy number and promoter efficiency.^121^ Common promoter types include (i) inducible promoters, which allow controlled gene expression based on environmental triggers. The Nisin-Controlled Gene Expression (NICE) system is one of the most used quorum-sensing systems, but its efficiency varies among *Lactobacillus* strains.^122,123^ Other inducible systems include P170, PxyIT, P­(zn)­zitR, SICE, Zirex, and ACE.^124^ (ii) Constitutive promoters, providing continuous protein expression. The pSIP system, derived from bacteriocin sakacin A/P operons, has been successfully used in *Lactobacillus reuteri*, *L. plantarum*, and *Lactobacillus gasseri*.^125–129^ While plasmid systems enable rapid protein expression, they pose challenges, such as plasmid instability, host metabolic burden, and potential gene loss. Synthetic biology advancements are improving plasmid stability by optimizing copy number regulation, metabolic load balancing, and AI-guided genetic modifications.^120^

#### 4.2.2 Genomic Integration Strategies

Regular genomic integration techniques provide permanent gene expression for long-term stability, ensuring that recombinant genes remain stable without selective pressure. Common methods include (i) Insertion Sequences (IS) and Phage Integration Systems, i.e., using IS elements or bacteriophage-derived sequences for stable chromosomal modifications, though limited by host genome compatibility,^130^ (ii) homologous recombination—the pSA3-based suicide vector (pTRK327) has facilitated stable genomic insertions in various *Lactobacillus* species,^131^ and (iii) temperature-sensitive plasmid vectors—allowing site-specific chromosomal gene integration with replication controlled at 35 °C (active) and 42 °C (inhibited), ensuring stable recombination.^132,133^ 
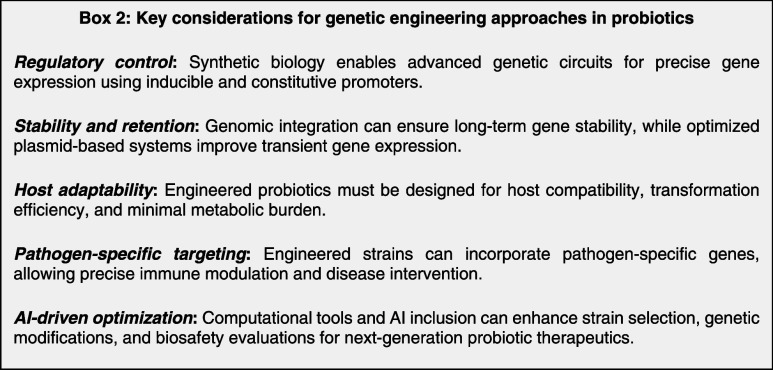


Although genomic integration methods improve gene retention and reduce plasmid-associated metabolic stress, they require optimized transformation protocols to enhance the efficiency. Synthetic biology enables the precise control of genome modifications, facilitating site-specific genetic alterations for improved therapeutic performance.^120^ Integrating synthetic biology and genetic engineering can transform probiotic-based drug delivery by enabling highly targeted, stable, and responsive therapeutic interventions. By leveraging advanced expression systems, genome editing techniques, and AI-guided optimization, engineered probiotics may evolve into versatile LBTs with significant clinical potential (see Box 2 for key considerations).^134–138^

### 4.3 Probiotic Surface Engineering

Payloads attached to the bacterial surface are one of the key methods for bacteria-driven drug delivery. The process of anchoring is crucial for the successful delivery of a therapeutic agent to the targeted tissue or cell. However, improper attachment methods and a dynamic tissue microenvironment may alter the surface properties of the outer membrane of the bacteria and affect the mobility of the bacterial vectors, resulting in off-target NP delivery. To provide enhanced attachment for NPs on bacterial surfaces, bacterial strains have been modified physiologically (magnetic, electrostatic, hydrophobic interactions, covalent attachment, biotin–streptavidin, and antigen–antibody) (Figure 7).^155–159^ Previous reports suggest that positively charged surfaces have a higher tendency of nonspecific attachment to a bacterial surface due to a net negative charge on most bacterial surfaces.^160^ In addition to surface charge, the mechanical stiffness of viscoelastic materials also plays a crucial role in the bacterial attachment yield. It has been shown that stiffer elastic materials (with an elastic modulus of 100 MPa) have a higher attachment rate than softer materials.^161,162^ The use of positively charged polyelectrolyte-layered microstructures as drug-delivering cargo enables the modulation of physiological properties on bacterial-driven surfaces, optimizing bacterial attachments.^163^ However, the concept of nonspecific interactions, including pH, ionic strength, and protein interactions through physical and electrostatic binding, creates limitations that hinder the efficacy of the therapeutic payload. To avoid these complications, various conjugation strategies have been explored.

**Figure 7:**
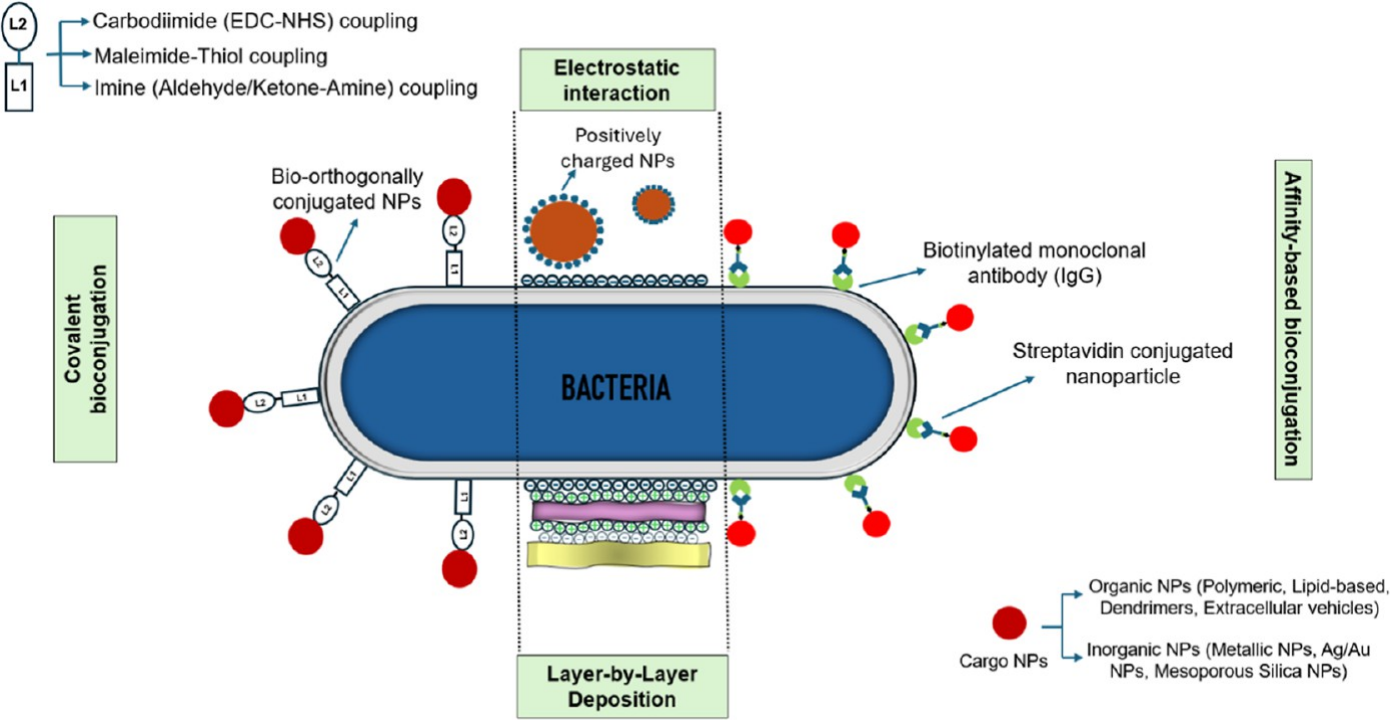
Different methodologies of conjugation of NPs on the bacterial surface for drug delivery applications. The most utilized methods for NP conjugation on bacterial surfaces are covalent bioconjugation, electrostatic interaction, layer-by-layer deposition, and affinity-based bioconjugation. Some standard covalent conjugation-based techniques (top left) and types of NPs as potential drug-delivering cargo (bottom right) have been highlighted.

The interaction between streptavidin and biotin is one of the most substantial noncovalent interactions and a paradigm for protein–ligand interactions.^164^ This coupling method for conjugating the NPs to the bacterial surface membrane allows the specific integration of micro/nanoparticles onto viable bacteria without harsh chemical/physical processes. It primarily includes quick incubation steps, followed by washing steps to remove unintegrated NPs. Several click-chemistry-based approaches are also employed for covalent bioconjugation of NPs to the bacterial surface because of their selectivity and high conjugation yield.^165,166^ One of the bioorthogonal ligands (L1) must be attached to the outer membrane of the bacteria (Figure 7). Conversely, the NPs can be surface functionalized with the other ligand (L2), which could then be bioconjugated with L1. Highly specific covalent ligand binding has also been employed for attaching the payload to bacterial surfaces, such as the use of the carbodiimide method, which includes protein coupling without affecting the carboxyl group of the second protein. Recent reports show that PLGA NPs encapsulated with perfluorohexane were conjugated to the peptidoglycan-rich surface of *Bifidobacterium longum* through carbonyl amide bonding.^167^ This method enabled longer retention of NPs and targeted delivery via bacteria, leading to precise tumor therapy. Another study investigated the controlled release of the chemotherapeutic drug Doxorubicin at tumor sites using a bioconjugation technique involving *E. coli* 1917 as a bacterial vector and polymer.^168^ Amphiphilic copolymers were immobilized via acid-labile linkers, and a poly­(ethylene glycol) copolymer was conjugated on the bacteria’s surface using a selective biorthogonal tetrazine/norbornene clicking reaction. The acid-labile linker 2-propionic-3-methylmaleic anhydride facilitated the specific conjugation. Results indicated controlled drug release at lower pH over 36 h, enhancing bacterial colonization and demonstrating increased antitumor efficacy, including tumor shrinkage and apoptosis induction in vivo in tumor-bearing mice.^168^

Innovative assembly designs have been developed to integrate nanostructures into bacteria to precisely deliver therapeutic payloads. For instance, bacteriabots guided by magnetic field gradients have significantly improved the navigation of biohybrid drug delivery systems, increasing bioavailability at target sites.^98,163^ The fabrication of bacteriabots often involves bioadhesives, ensuring that drug-encapsulated NPs remain near diseased cells or tissues. In one study, researchers employed a lectin-mannose anchoring system, leveraging the presence of type I pili (fimbriae I) on *E. coli* strains, which contain mannose-binding lectin groups.^169^ Another notable approach demonstrated a photoreversible NP cargo attachment to bacterial surfaces, controlled via light (red to far-red/infrared light ranging 650–1350 nm).^170^ Researchers engineered the *E. coli* membrane to present the photo switchable protein Phytochrome B (PhyB) via biotin–streptavidin coupling, enabling its interaction with phytochrome interaction factor 6 (PIF6), functionalized on polystyrene NP surfaces in this study. These advanced strategies promise further refinement using more precise molecular moieties, paving the way for enhanced targeted drug delivery applications with probiotics.

## 5 Delivery Routes and Formulation Approaches

Oral delivery of probiotics is the most promising approach for therapeutic delivery due to their ability to modulate gut microbiota. The oral-based probiotics treatment approach has been researched for various disease models, including colorectal cancer, liver diseases, and inflammatory bowel disease.^171^ Currently, probiotics are primarily used as supplements in commercial products and in a few clinical trials as freeze-dried powders and encapsulated oral capsules administration. However, challenges still exist for probiotic bacteria to perform effectively *in vivo* through oral administration because of the complex nature of the gut microenvironment. A gut microenvironment with low pH, presence of gastric juices, and gut microbiome interactions can inhibit bacteria survival, thus further leading to the limited colonization and proliferation of probiotics in the GI tract.^172,173^ Various engineering methods have been investigated for shielding probiotics with biomaterials or other biological functional motifs to improve their viability and therapeutic efficacy.

Microencapsulation materials such as polysaccharides (e.g., alginate) have been widely used for encapsulating probiotics due to their ability to gel-formulate via ionic cross-linking, pH-responsive properties, and biocompatibility. In a study, Chu et al. reported that using sodium alginate/protamine shells to encapsulate *L. casei* increased the survival rate of the bacteria by about 60 times compared to conventional sodium alginate beads.^174^ In another study, the researchers used layer-by-layer electrostatic self-assembly deposition of *E. coli Nissle* using positively charged chitosan and negatively charged alginate that resulted in providing enhanced protection of the probiotics compared to the clinically standard coating material, Eudragit L100-55.^146^ Microencapsulation of probiotics could also be an effective method to protect the probiotics from gastric acid and the bile salt environment. Recent research shows a unique design of thiolate oxidized konjac glucomannan (sOKGM) microspheres with pH responsiveness and mucoadhesive properties to encapsulate the probiotics *L. lactis* NZ9000.^175^ The microsphere-encapsulated probiotics displayed an enhanced survival rate in simulated gastric fluid with respect to that of bare probiotics. These studies indicate a robust method of microencapsulation of probiotics for oral drug delivery.

In addition to encapsulating probiotics with chemically synthesized and naturally derived biomaterials, a surface coating strategy for probiotics has also been employed to address low bioavailability and inadequate retention of oral probiotics in the gut. Lipids, due to their innate characteristics, including biocompatibility and biodegradability, are commonly used for coating/encapsulating probiotics. In a study, dioleoylphosphatidic acid (DOPA) and cholesterol were used to coat the surface of *E. coli Nissle* via biointerfacial supramolecular self-assembly. The lipid-coated probiotics displayed significant improvement in the survival rate, unchanged viability, and bioactivity in harsh environmental conditions, exhibiting enhanced efficiencies in oral delivery in treatment of colitis.^176^ Silk fibroin has also been used as a strong nanocoating agent for coating *E. coli Nissle* by assembling into β-sheet conformation, forming a shell structure to shield the probiotic bacteria against the harsh conditions of the GI tract as well as providing synergistic anti-inflammatory results.^177^ This nanocoating significantly enhanced the oral bioavailability and therapeutic efficacy of the probiotics, enabling sustained drug release and resistance against gastric and enzymatic degradation (up to 52-fold survival in simulated gastric fluid). The bacterial viability is unchanged (with a 91.5% coating efficiency), and intestinal colonization is enhanced up to 5.8-fold, reflecting the synergistic improvement in the anti-inflammatory outcomes. In another study, a high molecular-weight hyaluronan (HMW-HA) functionalized metal-phenolic network (MPN)-based layer-by-layer nanocoating was used to coat *E. coli Nissle* 1917, demonstrating ultraresistance to harsh gastrointestinal conditions, preferential adhesion and colonization in the inflamed colon, responsive degradation of the nanocoating under inflammatory conditions, and reshaping dysbiosis of intestinal bacteria for synergistic improvement of IBD lesions.^178^ An alternative yet appealing approach, implemented by Song et al., utilizes engineered bacterial spores within spore-coated nanomaterials to anchor the surface membranes of various probiotic strains, thereby offering protection from harsh stomach conditions^179^ (Figure 8). Bacterial spores containing calcium ions (Ca^2+^) provide anti-inflammatory effects by repairing the epithelial barrier and inhibiting pro-inflammatory factors such as IL-1β, TNF-α, and IL-6. When administered orally to DSS-induced colitis mice, these coated probiotic spores effectively colonized the colon, withstanding harsh gastric acids and enhancing commensal microbiota regulation.

**Figure 8:**
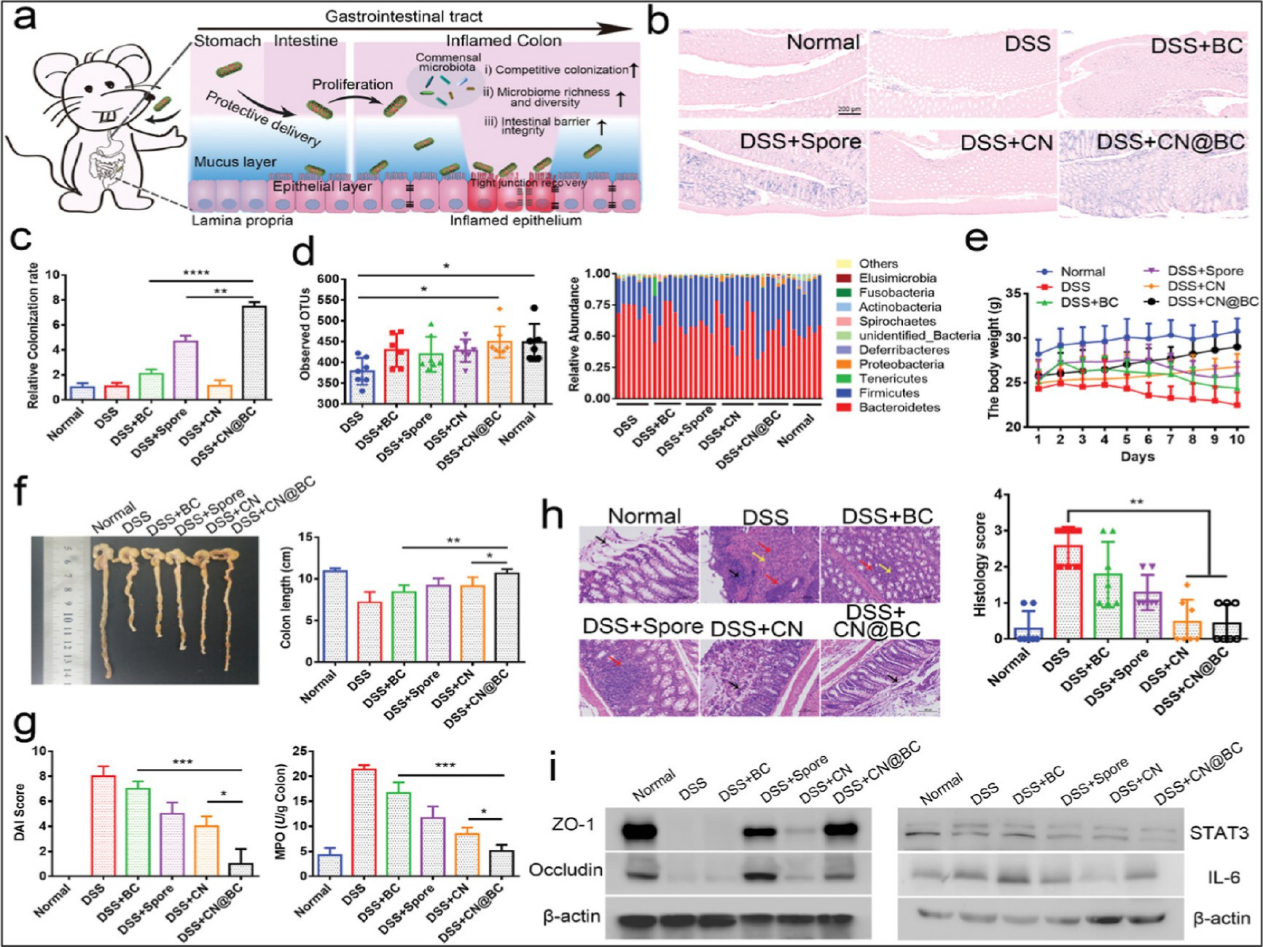
Assessment of the therapeutic effect of spore coat nanomaterial (CN@BC) in the colitis mouse model. (a) The schematic of bioinspired CN-coated probiotics for colitis treatment. (b) Representative Gram staining images and (c) semiquantitative analysis of microbiota number of the intestinal tissues harvested from the colitis mice after being treated with different groups with 1 × 10^7^ CFUs of probiotics. (d) Estimation of microbial community observed OTUs richness and relative abundance of gut microbiota (*n* = 7). (e) Daily body weight changes in each group during the treatment (*n* = 7). (f) Representative photos of colorectal specimens and the colon length measurement of each mouse after treatment (*n* = 7). (g) The DAI score and MPO were evaluated in the groups of Normal, DSS, DSS + BC, DSS + Spore, DSS + CN, and DSS + CN@BC, respectively (*n* = 7). (h) Representative H&E images of colorectal tissues in different groups (black arrow: necrotic cell debris, red arrow: lymphocyte and neutrophil infiltration, yellow arrow: connective tissue proliferation); and the histopathological score was recorded in each group after treatment (*n* = 7). (i) Western blotting analysis of ZO-1, Occludin, IL-6, and STAT3 upon the different treatments (*n* = 3). Data are presented as mean ± SD. Statistical significance was analyzed via one-way ANOVA with a Tukey posthoc test. *P* values: *P < 0.05, **P < 0.01, and ***P < 0.001.^179^ Copyright ©. Reprinted (Adapted) with permission from Qingling Song, Hongjuan Zhao, and Cuixia Zheng et al. *Advanced Functional Materials* **2021**.

Conversely, the core biomimetic NPs can be coated with the LTMs. These camouflaged NPs would be capable of bypassing the harsh GI tract environment while retaining the integrity of the synthetic NPs. Probiotic OMVs have been utilized as a coating material and can be employed for applications in immune modulation, cancer therapeutics, and inflammatory GI tract disorders.^180^ In a relevant study, *E. coli Nissle*-1917-derived OMVs were surface-coupled on the aldehyde silica microspheres (SAP).^181^ These OMVs derived from the probiotics were employed to mimic the probiotic surface membrane and its interactions. SAP@OMV microspheres significantly improved the survival of the mouse (DSS-induced acute colitis model), alleviated the harmful effects of the DSS by maintaining the colon length, reducing the colon injury and downregulating the expression of the inflammatory factors such as TNF-α and IL-1β, and increased the expression of the tight junction protein gene zonula occludens-1 (ZO-1). Similarly, in another study, the OMVs of the probiotic *E. coli Nissle1917* (EM) were utilized to encapsulate the curcumin-loaded mesoporous polydopamine NPs (MDPA@Cur).^182^ MPDA@Cur@EM released a very small amount of loaded Curcumin after incubation in simulated gastric fluid (SGF) and simulated intestinal fluid (SIF) for 48 h, indicating good resistance to harsh gastrointestinal conditions. The TEM observation further demonstrated that the architecture of MPDA@Cur@EM remained intact after incubation in SGF and SIF for 6h. Additionally, after the oral administration, the intestine segments from MPDA-cy7@EM treated DSS-colitis mice exhibited the strongest fluorescence signal and retained more in the inflamed lesions, suggesting efficient targeting. Consequently, MPDA@Cur@EM efficiently attenuates the inflammatory reaction and restores intestinal barrier functions, demonstrated by the multipronged manner of restoring redox balance, remodeling immune homeostasis, and reshaping the gut microecology.

Oral delivery remains the primary method for administering probiotics. Other than oral delivery, the scope for intranasal delivery of engineered probiotic bacteria for targeting respiratory viral infections has also been investigated.^183–186^ Furthermore, there is also growing interest in delivering the living/engineered probiotic bacteria and their components using microneedle patches for long-lasting antibacterial effects.^187–190^ Therefore, further interdisciplinary research on enhancing probiotic resilience and promoting beneficial gut microbiome interactions is essential for advancing probiotic therapy.

## 6 Challenges and Future Outlook

The field of probiotic drug delivery is rapidly evolving with several promising directions in therapeutics. The integration of CRISPR-based gene editing and other synthetic biology approaches will allow for the development of designer probiotics capable of sensing, responding to, and treating specific disease conditions in a personalized manner. AI and machine learning could further optimize strain selection, metabolic pathway engineering, and delivery strategies. Enhancing the ability of probiotic bacteria to selectively home to diseased tissues, such as hypoxic tumor microenvironments or inflamed intestinal regions, will be critical for improving therapeutic efficacy. Incorporating magnetically responsive NPs or chemically guided targeting mechanisms may allow precise control over probiotic drug carriers in vivo. Surface coatings, NP conjugation, biofilms, and biomaterial-based encapsulation strategies can protect probiotics from harsh gastric conditions and immune clearance while enabling controlled drug release at target sites. Smart biomaterials that respond to environmental cues, such as pH, temperature, or enzyme activity, could further enhance therapeutic efficiency. Continued innovation in probiotic engineering may unlock therapeutic functionalities far beyond current capabilities. Drawing on advances in synthetic biology, nanomedicine, and digital health, several prospective strategies, outlined below, illustrate how next-generation probiotics could integrate responsive control, precision targeting, and adaptive safety features. While these concepts are currently speculative, they provide a forward-looking framework for guiding research and translational efforts in the field (Box 3).
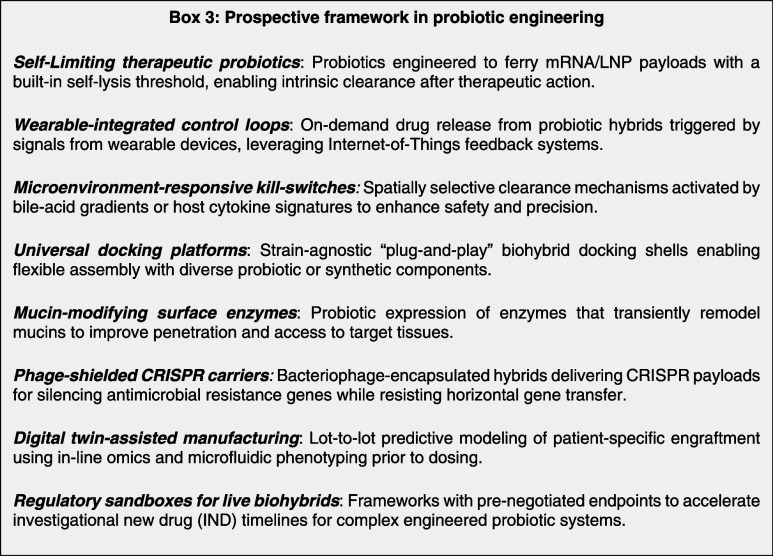


Various strategies have been explored to employ probiotics as NP carriers. However, significant challenges remain regarding the safety, biocompatibility, and overall metabolic and immunological effects of these carriers. Comprehensive evaluation of the associated factors is critical for their clinical translation. Notably, animal studies have demonstrated that oral exposure to nanomaterials can inhibit probiotic proliferation, trigger inflammatory responses in the gut immune system, promote opportunistic infections, and disrupt the composition and structural integrity of the gut microbiota.^191^ Longitudinal studies and clinical trial studies must be established to study the long-term effects on the gut microbiome from the use of NPs, especially metal-based NPs employed for photothermal effects in cancer and magnetic steering of probiotics toward the target. NP conjugation on probiotics has also been explored for drug delivery. Probiotic stability and functionality can be affected during formulation and oral delivery. The influence of nanoparticle parameters, including size and drug loading kinetics, particularly on NP conjugated genetically modified strains, should be systematically investigated to minimize disruptions to key functions such as therapeutic protein expression.

Microencapsulation of probiotics has been employed to improve the survival of probiotic bacteria for processing and gut translocation during delivery. Materials such as polysaccharides are used in high quantities for the coating of probiotics. Polysaccharide materials like chitosan, alginate, pectin, and others are used for encapsulation.^192^ Despite their biocompatibility for coating probiotics, their scalability with respect to the coating size and uniformity remains a challenge. Further emphasis on critical research for scalability and improvement of polysaccharides with bioactive functionality for encapsulation is needed.

To enhance the probiotics functionality for the “bug as a drug” approach, they are genetically modified using several gene editing techniques. However, the long-term safety of live genetically modified probiotics is not fully understood. Advances in nonreplicative bacterial vectors and bacterial ghost systems may offer safer alternatives for clinical use. Robust safety assessments must address potential pathogenicity, unintended metabolic activity, horizontal gene transfer, and host immune responses.^193^ In particular, the risk of transferring antibiotic resistance genes is a major concern given the global rise in antimicrobial resistance. While probiotic-based therapies hold great promise, their clinical translation will require standardized regulatory frameworks to evaluate safety, efficacy, and long-term effects.

Several critical attributes must be addressed to enable the clinical translation of probiotics as drug delivery systems. Potency should be defined through mechanism-linked assays, such as target cargo delivery, enzymatic flux, or quorum-triggered lysis, rather than viability alone. Robust biocontainment strategies, including auxotrophy, kill-switches, and nonmobilizable resistance elements, are essential to minimize ecological and safety risks. Clinically, interpatient microbiome variability complicates dose–response relationships, prompting interest in adaptive trial designs and companion diagnostics. Additional Chemistry, Manufacturing, and Controls (CMC) requirements arise from the dual-spec compliance of a living carrier and a synthetic cargo or shell, particularly in combinatorial approaches. Changes in fermenter conditions, cryoprotectants, or conjugation chemistry can alter probiotic function, making scale-up and comparability challenging. Intellectual property and nomenclature considerations also present hurdles, requiring unambiguous strain designation and clear freedom-of-operate around genetic parts, linker chemistries, and delivery devices. Probiotics as biohybrid carriers have great potential for developing next-generation drug delivery systems.
